# Iron necessity for chlamydospore germination in *Fusarium oxysporum* f. sp. *cubense* TR4

**DOI:** 10.1007/s10534-023-00519-4

**Published:** 2023-06-29

**Authors:** Evans Were, Altus Viljoen, Frank Rasche

**Affiliations:** 1 Institute of Agricultural Sciences in the Tropics (Hans-Ruthenberg-Institute), University of Hohenheim, 70599 Stuttgart, Germany; 2 Department of Plant Pathology, Stellenbosch University, Private Bag X1, Matieland, 7602 South Africa

**Keywords:** Germ tube, Outgrowth, Polarized, Ribonucleotide reductase, Quiescence

## Abstract

Fusarium wilt disease of banana, caused by the notorious soil-borne pathogen *Fusarium oxysporum* f. sp. *cubense* Tropical Race 4 (Foc TR4), is extremely difficult to manage. Manipulation of soil pH or application of synthetic iron chelators can suppress the disease through iron starvation, which inhibits the germination of pathogen propagules called chlamydospores. However, the effect of iron starvation on chlamydospore germination is largely unknown. In this study, scanning electron microscopy was used to assemble the developmental sequence of chlamydospore germination and to assess the effect of iron starvation and pH *in vitro*. Germination occurs in three distinct phenotypic transitions (swelling, polarized growth, outgrowth). Outgrowth, characterized by formation of a single protrusion (germ tube), occurred at 2 to 3 h, and a maximum value of 69.3% to 76.7% outgrowth was observed at 8 to 10 h after germination induction. Germination exhibited plasticity with pH as over 60% of the chlamydospores formed a germ tube between pH 3 and pH 11. Iron-starved chlamydospores exhibited polarized-growth arrest, characterized by the inability to form a germ tube. Gene expression analysis of *rnr1* and *rnr2*, which encode the iron-dependent enzyme ribonucleotide reductase, showed that *rnr2* was upregulated (p < 0.0001) in iron-starved chlamydospores compared to the control. Collectively, these findings suggest that iron and extracellular pH are crucial for chlamydospore germination in Foc TR4. Moreover, inhibition of germination by iron starvation may be linked to a different mechanism, rather than repression of the function of ribonucleotide reductase, the enzyme that controls growth by regulation of DNA synthesis.

## Introduction

Chlamydospores are hardy thick-walled asexual spores produced by diverse fungi, including *Fusarium oxysporum* f. sp. *cubense* (Foc), the notorious root-infecting pathogen causing Fusarium wilt disease of banana (Viljoen et al. 2020). *Fusarium oxysporum* f. sp. *cubense* Tropical Race 4 (Foc TR4), is considered the most virulent and devastating race of Foc. Foc TR4 is spreading inexorably and poses a threat to the food security and income of nearly 400 million people that depend on banana (Zheng et al. 2018; Viljoen et al. 2020; van Westerhoven et al. 2022). Chlamydospores are the primary source of Foc inoculum and can remain quiescent in infested soils for decades. When suitable conditions are encountered, chlamydospores in soil undergo a revival cellular process called germination (Pegg et al. 2019; Were et al. 2022a). Chlamydospore germination is a crucial step in the infection of host roots and development of Fusarium wilt (Pegg et al. 2019). Fusarium wilt can be suppressed by targeting chlamydospore germination through the manipulation of soil pH to 7.0 or close thereto, and by reduction of the bioavailability of iron through application of iron chelators (Peng et al. 1999; Dita et al. 2018; Segura-Mena et al. 2021). However, the mechanisms of inhibition and the distinct role of iron and pH on chlamydospore germination are largely unknown.

Quiescence in fungal spores is a non-proliferative cellular state of reversible cell cycle arrest (Rittershaus et al. 2013; Blatzer and Latgé 2021). Quiescence is characterized by reduced metabolic activity and accumulation of storage molecules, such as trehalose (Wyatt et al. 2013; Hayer et al. 2014). The transition from quiescence to germination generally occurs in three distinct phenotypic transitions: dormancy to swelling (isotropic increase in size), swelling to polarized growth, and the emergence of a germ tube (outgrowth) (Sephton-Clark and Voelz 2018). Outgrowth marks the end of the germination process. It is considered the first step in the formation of a fungal colony and a key developmental stage in the life cycle of fungi (Sephton-Clark and Voelz 2018). Spore germination has been widely investigated in fungal pathogens (Hayer et al. 2014; Turgeman et al. 2016). However, spore germination in Foc TR4 is largely unknown. Moreover, most studies on spore germination in Foc have been conducted using conidia (Li et al. 2011; Meldrum et al. 2013; Deng et al. 2015). However, conidia of *Fusarium oxysporum* may not be appropriate substitutes for chlamydospores. This is because both types of spores are typically formed under distinct environmental conditions, perform distinct functions in the fungus’s life cycle, and exhibit different degrees of persistence in soil (Ohara and Tsuge 2004; Ohara Leslie and Summerell 2006).

Spore germination is marked by heightened metabolic activity during which biomolecules are synthesised to rebuild hyphae from the disintegrating spore (Deng et al. 2015; Sephton-Clark and Voelz 2018; Balotf et al. 2021). For example, outgrowth in filamentous fungi occurs after nuclear division which, in turn, is dependent on DNA synthesis (Cohen et al. 2019; Greene et al. 2020; Steenwyk 2021). The synthesis of DNA requires ribonucleotide reductase (RNR), the iron-dependent enzyme which converts ribonucleotides to deoxyribonucleotides (dNTPs) which are the precursors for DNA synthesis and repair (Greene et al. 2020; Steenwyk 2021). In *F. oxysporum*, RNR has two non-identical homodimeric subunits, a large subunit Rnr1 and a small one-Rnr2, which are encoded by the genes *rnr1* and *rnr2*, respectively (Cohen et al. 2019). The Rnr1 subunit contains the catalytic site and allosteric sites that control enzyme activity and specificity (Greene et al. 2020). On the other hand, the Rnr2 subunit contains a non-heme iron centre, which generates a tyrosyl free radical that is essential for catalysis.

Iron acquisition is crucial for the survival and growth of invading microorganisms. In *Fusarium oxysporum*, iron homeostasis is regulated by the basic leucine zipper (bZIP) transcription factor, HapX (López-Berges et al. 2012a, b). Under conditions of iron starvation, HapX is activated, resulting in the synthesis of low-molecular-weight (< 1 kDa) secondary metabolites called siderophores, that are essential for iron scavenging and storage (Hider and Kong 2010). Although several siderophores, including ferrichrome, fusigen, and fusarinine, have been identified in Foc (Anke et al. 1973; Beckmann et al. 2013; Were et al. 2022a, b), the mechanisms of siderophore-mediated iron transport remain largely unknown.

Iron is an indispensable micronutrient for all eukaryotic organisms and an essential cofactor of RNR (Furukawa et al. 1992; Colombo et al. 2014; Fukada et al. 2019). Given the significance of iron, we hypothesized that iron is fundamental for outgrowth in Foc TR4 and that iron scarcity leads to selective optimization of RNR function by enhanced expression of *rnr1* and *rnr2* genes. Accordingly, the specific objectives were to assess (i) the effect of iron starvation on outgrowth and the expression of *rnr1* and *rnr2* genes, and (ii) the effect of extracellular pH on outgrowth.

## Materials and methods

### Fungal strain, culture conditions and production of chlamydospores

*Fusarium oxysporum* f. sp. *cubense* Tropical Race 4 (TR4) VCG 01213/16 was obtained as a frozen stock on 30% (*v*/*v*) glycerol from the Department of Plant Pathology Stellenbosch University, South Africa. The isolate was preserved at −80 °C and revived by culturing on potato dextrose agar (PDA) at 28 °C for 5 days. All chemicals, unless otherwise stated, were purchased from Carl Roth (Karlsruhe, Germany).

### Chlamydospore germination time-course assay

Chlamydospores were produced from the culture and purified as previously described (Goyal et al. 1973; Were et al. 2022b). A time-course experiment was conducted to determine the developmental sequence of germinating chlamydospores of Foc TR4. The experiment was conducted in 24-well culture plates (Costar, Cambridge, MA, USA) using Barz broth (Were et al. 2022b). Samples for phenotypic analysis of germination were retrieved after 30 min and thereafter every hour for duration of 10 h post induction of germination (h.p.i). Samples were retrieved in 2-mL tubes, chilled on ice, and centrifuged at 13,000 r.p.m. for 10 min at 4 °C (Eppendorf 5810R Centrifuge, Eppendorf, Hamburg, Germany). Chlamydospores were washed with ice-cold cell wash buffer (PBS-T: 137 mM, NaCl; 2.7 mM, KCl; 2 mM, KH_2_PO_4_; 0.005% *v/v*, Triton X-100; pH 7.4) by vortexing at maximum speed for 1 min, followed by centrifugation at 13,000 r.p.m. for 10 min at 4 °C. This procedure was repeated using sterile deionized water.

A developmental sequence of germinating chlamydospores was assembled by bright-field microscopy using a LeicaDM750 microscope equipped with a Leica ICC50 HD camera (Leica, Heerbrugg, Switzerland). Germinated, polarized, and round chlamydospores were counted using a haemocytometer and a Zeiss Axioskop microscope (Carl Zeiss, Jena, Germany). Micrographs were captured using a LeicaDM750 microscope, converted to 8-bit grayscale or 24-bit RGB, and annotated using CorelDraw 12.0 (Corel, Ottawa, Canada). This procedure allowed for the monitoring of the progression of chlamydospore germination.

### Scanning electron microscopy (SEM)

Scanning electron microscopy (SEM) was used to obtain ultra-structural details of germinating chlamydospores. Chlamydospore specimens for SEM analysis were prepared as described previously (Were et al. 2022b). Specimens were visualized with a Zeiss Merlin scanning electron microscope (Carl Zeiss) with a Gemini-type field emission gun electron column (FEG-SEM) equipped with two Oxford Instruments X-MaxN 150 SDDs. Typical imaging conditions were magnification of 1–3 × 10^4^, a working distance of 5–10 mm, 2–3 kV, a beam current 100–200 pA and using an In Lens secondary electron detector. Micrographs of specimens were captured in TIF format using a pixel averaging noise reduction algorithm and SmartSEM software (Carl Zeiss).

### Cellular metabolic activity and germination in iron-starved chlamydospores

The effect of iron-starvation on chlamydospore germination was assessed by determining the percent germination and cellular metabolic activity of iron-starved chlamydospores. Iron starvation was induced using 2,2′-dipyridyl, a synthetic lipophilic iron chelator that sequesters both extracellular and intracellular Fe^2+^ and Fe^3+^ pools (Breuer et al. 1995; Romeo et al. 2001; Asai et al. 2022). A stock solution of 2,2′-dipyridyl (20 mM) was prepared in dimethyl sulfoxide (DMSO), filter-sterilized using a 0.22 µm syringe filter (Sartorius, Göttingen, Germany), and stored in the dark at 4 °C.

Cellular metabolic activity was determined using the Alamar Blue kit (Bio-Rad, Hercules, CA, USA), that measures activity of the mitochondrial respiratory chain as a readout of cell viability. The assay was conducted in Barz medium supplemented with different concentrations of 2,2′-dipyridyl [1, 10, 100, 200 µM and control (10% *v*/*v* DMSO)] by following the manufacturer’s protocol (Bio-Rad, Hercules, CA, USA). Absorbance was measured using a microplate reader (Tecan, Maennedorf, Switzerland). Absorbances were normalized to the DMSO control, where cell viability was set to 100%. The effect of iron-starvation on chlamydospore germination was conducted in 24-well plates as described earlier using Barz supplemented with 2,2′-dipyridyl [1, 10, 100, 200 µM and control (10% *v*/*v* DMSO)]. Plates were incubated for ten hours and germinated chlamydospores were determined as described earlier.

To further establish the effect of iron starvation on the chlamydospore germination process, a two-step experiment (iron starvation and iron repletion) was conducted. First, in the iron starvation experiment, chlamydospores (10^3^ per well) were incubated in a 24-well culture plate containing 900 µL of Barz broth per well supplemented with 2,2′-dipyridyl (100 µM). After 5 h, rounded, polarized, and outgrown chlamydospores was determined as described earlier. In the second, iron-replete experiment, chlamydospores from the iron-starvation experiment were washed as described earlier to remove 2,2′-dipyridyl and further incubated for 5 h in fresh Barz medium. Afterward, rounded, polarized, and outgrown chlamydospores was determined as described earlier.

### RNA extraction and cDNA synthesis

To analyse the expression of RNR genes (*rnr1* and *rnr2*), chlamydospore samples were collected from both iron-starvation and repletion experiments. The Chlamydospores were obtained by pooling suspensions from two wells and then transferred into 2-mL tubes. The samples were washed as described earlier. Then, samples were stabilized by immediately suspending in 500 µL of RNAlater RNA (Qiagen, Hilden, Germany) and stored at −80 °C until RNA extraction. Samples were retrieved and thawed on ice prior to extraction of total RNA. Total RNA was isolated from chlamydospores using the RNeasy kit following the manufacture’s protocol (Qiagen). RNA samples were subsequently reverse transcribed to cDNA using a QuantiTect Reverse Transcription Kit following the manufacture’s protocol (Qiagen).

### Quantitative RT-PCR

Reverse transcription quantitative polymerase chain reaction (RT-qPCR) was performed on a StepOne Real-Time PCR system (Applied BioSystems, CA, USA) using Power SYBR Green PCR Master Mix (Applied Biosystems) and gene-specific primer pairs (Table 1) (Cohen et al. 2019). All samples were amplified in triplicate using the following thermal cycling conditions: 95 °C for 20 s, 40 cycles of priming at 54 °C for 20 s, and elongation at 72 °C for 20 s. Cycle thresholds were obtained using the StepOne software (v2.0, Applied BioSystems, CA, USA). Relative gene expression levels were normalized to that of the *Fusarium oxysporum* actin-lateral binding (*ALb*) gene using the ΔΔCt method (Yuan et al. 2006).

**Table 1:** Primers used for analysis of the expression of ribonucleotide reductase genes (*rnr1* and *rnr2*) and actin-lateral binding (*ALb*) gene in chlamydospores of *Fusarium oxysporum* f. sp. *cubense* Tropical Race 4 (Cohen et al. 2019)

Target gene	Primer name	Sequence (5ʹ → 3ʹ)
*rnr1* (*F. oxysporum* ribonucleotide reductase large subunit)	rnr1-F	CATCAAGGCTGATGTTGAGG
	rnr1-R	CTTGACACCCAACTCTTCTTCC
*rnr2* (*F. oxysporum* ribonucleotide reductase small subunit)	rnr2-F	TACTTTGGATCCGGAAGCTG
	rnr2-R	TTTTCATCCACCCTGAGTCC
*ALb* (*F. oxysporum* actin-lateral binding protein)	ALb-F	GGTTTCCCTTCAGCCTTTTC
	ALb-R	CGGAGCTGGTTCATTTTCTC

### Statistical analyses

Data analysis and visualisation were performed using R (v.4.0.2; R Development Core Team 2020). Prior to statistical analysis, data was checked for normality and homoscedasticity (Kozak and Piepho 2018). Homoscedasticity of the data was verified using Levene’s test, whereas the normality the data was verified using the Shapiro–Wilk test and diagnostic plots (histograms and Q-Q plots). Comparisons were made between treatments and the control. Statistical significance was declared if p < 0.05. Significant differences between treatments and the control were tested using analysis of variance (ANOVA) and subsequent post hoc analysis using Tukey’s Honest Significant Difference (Tukey HSD) test. All data are expressed as mean ± standard error of the mean (Kozak and Piepho 2020).

## Results

### Germination of Foc TR4 chlamydospores is asynchronous

**Fig. 1:**
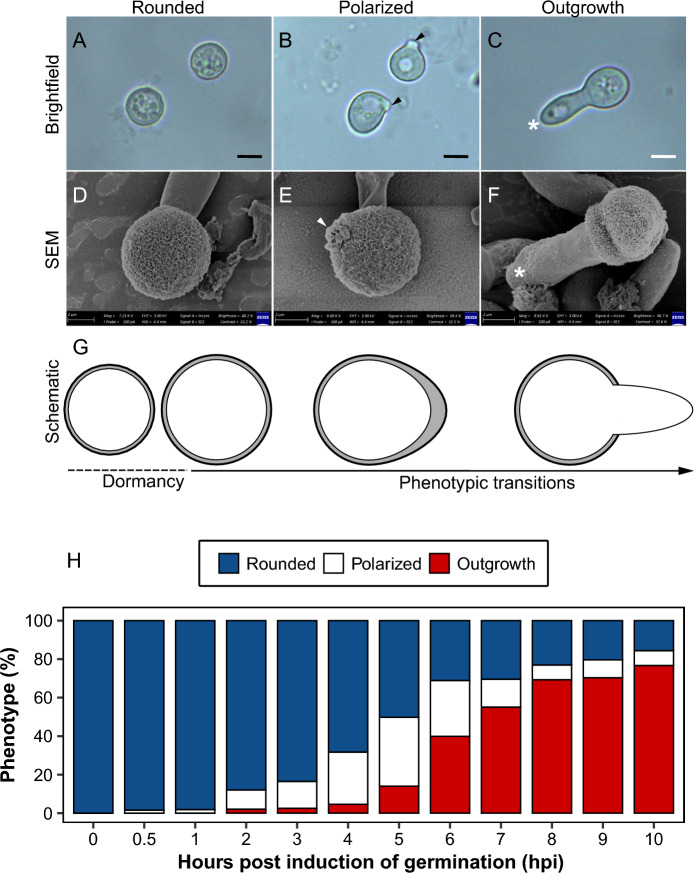
Bright-field and scanning electron microscopy micrographs showing the transitions during germination of chlamydospores of *Fusarium oxysporum* f. sp. *cubense* Tropical Race 4 (Foc TR4): rounded (**A**, **D**), polarized growth (**B**, **E**), and outgrowth  (**C**, **F**). The stages of germination are further illustrated in the schematic (**G**). The site of polarity establishment (indicated by arrow heads) on polarized chlamydospores, is the point where the germ tube (indicated by white asterisk) emerges during outgrowth. Scale bar = 20 µm. Phenotypic changes during the process of germination of chlamydospores in Foc TR4 (**H**)

Chlamydospores appeared round and enveloped in a double layered wall: a thin inner wall and a thick outer wall, when examined with brightfield microscopy (Fig. 1A). The outer wall was composed of distinctly fibrillar material, when examined by SEM (Fig. 1D–F). Germination was characterized by three distinct phenotypic transitions: swelling, to polarized growth, to outgrowth (Fig. 1A–H). Swelling occurred within 0.5 to 1 h post induction of germination (h.p.i), during which the rounded chlamydospores appeared larger than the dormant chlamydospores and possessed a highly granulated cytoplasm (Fig. 1A, G). At 2 to 3 h.p.i, deposition and concentration of cellular material was observed at a single specific point on the chlamydospore inner wall (Fig. 1B). This cellular material, whose molecular identity was not determined, appeared as a bright spot towards which growth was directed, thereby defining the spore front and domain. This domain was maintained, resulting in a polarized chlamydospore with a pear-shaped form (Fig. 1B, E). Outgrowth, the most conspicuous transition, was observed starting at 2 to 3 h.p.i and a maximum value of 69.3% to 76.7% chlamydospores produced a single protrusion (germ tube) between 8 to 10 h.p.i (Fig. 1C, F and H). At this point, chlamydospores were considered to be fully germinated.

Notably, the germ tube emerged from the chlamydospore by the extension of the inner spore wall after local rapture of the outer wall at the site of polarity establishment (Fig. 1B). The germ tube progressively grew into hyphae that extended along a polar axis (Fig. 1C). Importantly, swelling, polarized growth, and outgrowth occurred subsequently, but not simultaneously, as evidenced by the rapid remarkable transition that was observed in some chlamydospores. This observation suggests that chlamydospore germination in Foc TR4 is an asynchronous process (Fig. 1H).

### Iron is essential for metabolic activity and outgrowth in chlamydospores

**Fig. 2:**
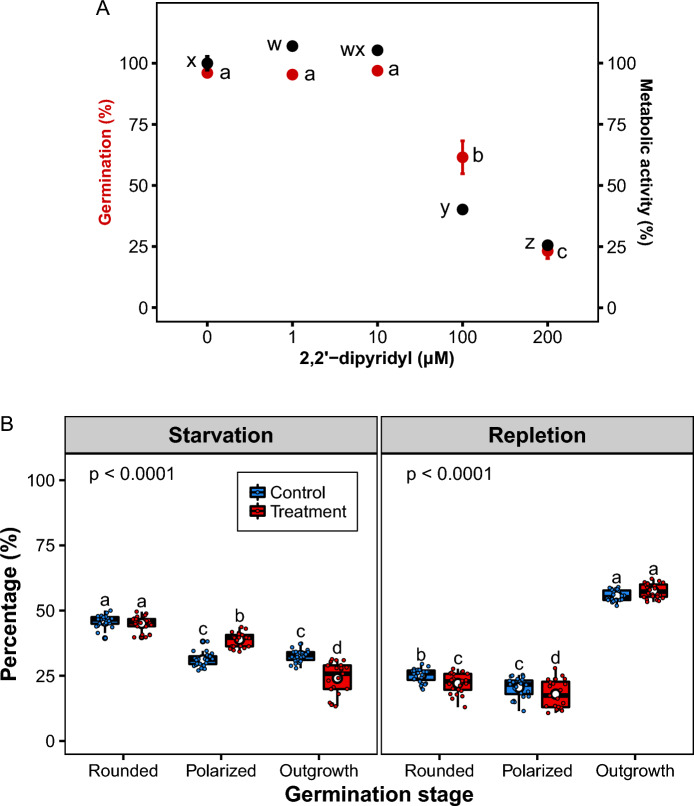
Effect of iron starvation on cellular metabolic activity and germination on chlamydospores of *Fusarium oxysporum* f. sp. *cubense* Tropical Race 4 (**A**, **B**). Boxplots show the upper and lower quartile, median (bold horizontal bar), mean (white circle), and whiskers (vertical lines)

At low concentration (1 and 10 µM) of 2,2′-dipyridyl, cellular metabolic activity of chlamydospores was stimulated, but decreased (p < 0.0001) as the concentration of 2,2′-dipyridyl increased (Fig. 2A). Similarly, chlamydospore germination decreased (p < 0.0001) as the concentration of 2,2′-dipyridyl increased (Fig. 2B). Iron-starved chlamydospores underwent through swelling but halted their developmental progression, thereby accumulating at polarized growth stage. The percentage of polarized chlamydospores in the treatment was 38.90 ± 0.54% compared to 31.11 ± 0.52 in the control (Fig. 2B). This polarized growth-arrest was characterized by the reduced inability to form a germ tube. Only 24.01 ± 1.24% of chlamydospores switched to outgrowth in the presence of 2,2′-dipyridyl, compared to 32.70 ± 0.49% in the control (p < 0.0001).

Chlamydospores, which exhibited polarized growth-arrest, contained perceptibly smaller and fewer cytoplasmic inclusions, indicating defects in replication or differentiation of cellular organelles. In marked contrast, control chlamydospores exhibited no discernible change in cytoplasmic inclusions and progressed normally through germination, resulting in the formation of a germ tube (outgrowth). Chlamydospores exhibiting polarized growth-arrest were rescued by iron repletion, resulting in restoration of germination (p < 0.0001) (Fig. 2B).

### Expression of the *rnr2* gene is up-regulated in iron-starved chlamydospores

**Fig. 3:**
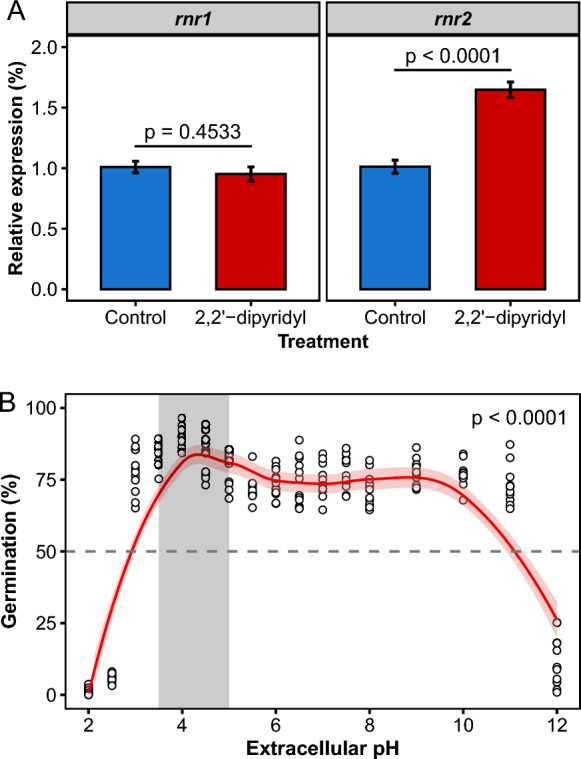
Effect of iron starvation on the expression of genes *rnr1* and *rnr2* that encode the Rnr1 and Rnr2 subunits, respectively, of the ribonucleotide reductase enzyme in chlamydospores of *Fusarium oxysporum* f. sp. *cubense* Tropical Race 4 (Foc TR4) (**A**). Effect of pH on chlamydospore germination in Foc TR4 (**B**). The dotted line denotes the 50% germination of chlamydospores

Expression of the *rnr2* gene was up-regulated in iron-starved chlamydospores, as compared with the control (p < 0.0001) (Fig. 3A). In contrast, expression of the *rnr1* gene did not differ between iron-starved chlamydospores and the control (p > 0.05) (Fig. 3A).

### Chlamydospore germination exhibits plasticity with extracellular pH

Chlamydospore germination was influenced by extracellular pH (p < 0.0001) (Fig. 3B). Germination was apparent over a wide pH range and exhibited double maxima; a primary peak (optimal) on the acid side from pH 3.5 to 4.5, where 75.3% to 94.3% of chlamydospores formed a germ tube. A secondary peak was noted close to neutrality between pH 8 and 9.5, where 67.8% to 84% of chlamydospores formed a germ tube (Fig. 3B). An increase of 0.5 pH units from pH 2.5 to 3.0 induced a 14-fold increase in germination (p < 0.0001). Notably, chlamydospores appeared small in Barz broth below pH 3.0 compared to chlamydospores above pH 10.0. Moreover, disintegration of incipient germ tubes was frequently observed below pH 3.0. Above pH 10.0, however, the produced germ tubes were often large with a small terminal globular structure, resembling a newly formed chlamydospore.

## Discussion

Chlamydospore germination is crucial in the infection of banana roots by the notorious fungal pathogen Foc TR4 and severity of Fusarium wilt disease (Dita et al. 2018; Pegg et al. 2019). Fusarium wilt can be suppressed by targeting chlamydospore germination through iron starvation caused by application of iron chelators or manipulation of soil pH, but the underlying mechanism of inhibition remains elusive (Peng et al. 1999; Segura-Mena et al. 2021). The findings of this study demonstrate that iron-deficient chlamydospores are unable to form a germ tube.

Consistent with previous work (Plante and Labbé 2019), we found that iron starvation results in polarized growth arrest and a concomitant decrease in cellular metabolic activity. Our data suggest that chlamydospore germination is a developmental process that requires iron. Spore germination in Foc TR4 entails resumption of metabolism and *de novo* synthesis of macromolecules to rebuild hyphae from the disintegrating spore (Deng et al. 2015). Macromolecular biosynthesis and energy generation require a unique enzymatic repertoire and bioavailability of cofactors, such as iron (Philpott et al. 2012). Polarized-growth arrest observed in Foc TR4 chlamydospores may reflect attenuation of cell cycle progression arising from a paucity of dNTPs available for DNA synthesis in iron-starved chlamydospores (Renton and Jeitner 1996; Cohen et al. 2019; Greene et al. 2020). DNA synthesis immediately precedes mitosis and subsequently nuclear division, which is indispensable for outgrowth in many fungi (Fukada et al. 2019). Nuclear division and migration of the daughter nucleus into the germ tube are contingent on mitosis control (Renton and Jeitner 1996; Cohen et al. 2019; Greene et al. 2020). Iron is required for assembly and activity of ribonucleotide reductase, an enzyme that catalyses the rate-limiting step in the production of dNTPs, the precursors for DNA synthesis (Cohen et al. 2019; Greene et al. 2020). Thus, the enhanced expression of *rnr2* suggests that iron scarcity leads to selective optimization of RNR function at the expense of other non-essential iron-dependent processes, to allow for DNA synthesis and repair. Similar findings have also been reported in *Saccharomyces cerevisiae* (Sanvisens et al. 2011).

Germination of Foc TR4 chlamydospores occurred over a wide pH range, which is consistent with studies in *F. oxysporum* (Cruz et al. 2019), *Harpophora maydis* (Degani and Goldblat 2014), and *Rhizopus delemar* (Turgeman et al. 2016). Maximum germination observed in the acidic and alkaline pH may denote different mechanism by which pH modulates germination. Specifically, the isoelectric point of protoplasmic proteins at this pH is at a low point, but increases under acidic and alkaline conditions (Caracuel et al. 2003; Turgeman et al. 2016). Moreover, interference with cellular processes, including protein synthesis, protein folding and therefore enzyme activity, cell wall remodelling and reduced availability of nutrients, are likely involved (Gaitanaki et al. 1990; Caracuel et al. 2003; Turgeman et al. 2016). Under field conditions, however, extreme acidic and alkaline conditions rarely occur. Nevertheless, from a biological perspective, our findings suggest a robust adaptation of Foc TR4 to ambient pH. Thus, from a virulence perspective, this might reflect the potential of Foc TR4 to infect plants in diverse soils.

The pH of the environment plays a crucial role in the infection process of *F. oxysporum* for instance, through the regulation of nutrient bioavailability and modulation of virulence factors such as fusaric acid (Gielkens et al. 1999; Fernandes et al. 2017; López-Díaz et al. 2018; Palmieri et al. 2023). *Fusarium oxysporum* relies on the highly conserved zinc finger transcription factor PacC and the PalH/Rim signalling pathway to sense and respond to extracellular pH (Caracuel et al. 2003). *In planta*, *F. oxysporum* secretes an array of effector proteins, which promote host colonization. For instance, a functional homolog of the plant regulatory peptide RALF (rapid alkalinization factor) efficiently induces host alkalinisation which, in turn stimulates MAPK-driven invasive growth (Fernandes et al. 2017; Mariscal et al. 2022).

Chlamydospore germination in Foc TR4 was characterized by the developmental sequence of three discrete morphological changes transitioning to swelling, polarized growth, and outgrowth. These transitions did not occur uniformly in a chlamydospore population, suggesting that the process of chlamydospore germination in Foc TR4 is asynchronous. Germination asynchrony may be a strategy for biological bet-hedging. It is a strategy employed by organisms to mitigate risk from unpredictable environments. Biological bet-hedging is particularly crucial for fungi that inhabit soils, which frequently experiences disturbances and fluctuations in temperature, humidity, and nutrient availability. (Stelkens et al. 2016), or may denote heterogeneity within a spore population (Wyatt et al. 2013). Asynchronous germination has also been reported in *Penicillium marneffei* (Zuber et al. 2003), *Saccharomyces paradoxus* (Stelkens et al. 2016), and *Cryptococcus neoformans* (Ortiz et al. 2021).

Phenotypic transitions observed in germinating chlamydospores of Foc TR4 are reminiscent of filamentous fungi, but differ in the timing between transitions (Sephton-Clark and Voelz 2018). This difference could reflect strain variability of the pathogen or distinct experimental systems. Chlamydospore swelling at the onset of germination suggests an increase in spore volume, which is typical of germinating fungal spores (Sharma et al. 2016; Hayer et al. 2014). Increase in spore volume results from hydration of the spore and is a fundamental step in the germination process (Hayer et al. 2014; Sephton-Clark and Voelz 2018). Hydration of the spore can be passive, following hydrolysis of trehalose to glucose (Sharma et al. 2016) or ATP-driven via aquaporins (Turgeman et al. 2016).

Polarized growth in filamentous fungi is well established (Ghose et al. 2021). A polar site is defined inside the cell (polarity establishment), from where growth is directed along a polar axis (polarity maintenance) (Ghose et al. 2021). Polarized growth is regulated by spatio-temporal activation of Rho-family guanosine triphosphatases (GTPases) and requires specialized GTP-binding proteins, called septins. Septins localize in a cortical region and serve as positional landmarks for activation and recruitment of the polarity machinery (Bonazzi et al. 2014; Ghose et al. 2021; Hall and Wallace 2022). This defines the cell’s front, thereby creating a polarity domain and protein complex, termed the polarisome (Ghose et al. 2021; Mishra et al. 2022). The polarisome is sustained by a constant supply of exocytic vesicles containing precursors for cell surface expansion and growth of the cell tip (Mishra et al. 2022). Vesicles aggregate at the cell tip and together with cytoskeleton components form the Spitzenkörper, a highly dynamic and pleomorphic complex that regulates hyphal growth and morphogenesis (Bonazzi et al. 2014; Ghose et al. 2021; Mishra et al. 2022). Hence, for now, it may be only speculated that the bright spot observed in polarized chlamydospores represents the polarisome, but further confirmative analysis will be necessary.

## Conclusion

Our results suggest that the process of chlamydospore germination in Foc TR4 is developmentally orchestrated and iron-dependent. Our findings highlight the role of iron and pH in the process of chlamydospore germination and suggest that in soil, disease suppression by manipulation of soil pH may act via other mechanisms besides the alteration of iron bioavailability. Although iron is crucial, the role of other elements like copper in chlamydospore germination and virulence of Foc TR4 cannot be ignored, especially given the impact of pH on their bioavailability. Therefore, it is necessary to investigate the importance and function of copper in these processes.
